# COVID-19 vaccination rates and related outcomes in adults with intellectual and developmental disabilities (IDD): An application of linked administrative health data to support Ontario’s COVID-19 response.

**DOI:** 10.23889/ijpds.v7i3.2065

**Published:** 2022-08-25

**Authors:** Natalie Troke, Diana An, Eliane Kim, Yona Lunsky, Robert Balogh, Lesley Plumptre

**Affiliations:** 1 ICES; 2 CAMH; 3 Ontario Tech University

## Objectives

Equitable vaccine distribution was essential in supporting Ontario’s COVID-19 response, particularly for persons with intellectual and developmental disabilities (IDD), who have been disproportionately impacted by the pandemic. An Applied Health Research Question (AHRQ) request through ICES, sought to report on COVID-19 vaccination uptake in adults with and without IDD.

## Approach

To examine the proportion of adults with IDD vaccinated for COVID-19, a request from the Ministry of Children, Community and Social Services (MCCSS) was approved by the ICES-AHRQ team. A secondary goal was to explore if vaccinated individuals with IDD experienced similar levels of protection from COVID-19 infection, to the general population. The open cohort was derived by linking vaccination events reported in Ontario COVID-19 Vaccine Data, between December 2020 to December 2021, to several administrative health databases. An algorithm was used to define IDD by a series of inpatient hospitalizations, emergency department and/or physician visits, with IDD diagnostic codes.

## Results

This exploratory study shows that Gypsy, Traveller and Roma young people are more likely to be disadvantaged across multiple dimensions such as health and housing, and are less likely to achieve level 1, 2 and 3 educational qualifications compared to their peers.

## Conclusion/Implications

Administrative health data and analytics through the ICES-AHRQ program were used in responding to COVID-19, and providing public health guidance, for those with IDD. To inform decision making and policy actions related to immediate vaccination strategies, MCCSS used such findings to target vaccination efforts in specific IDD subpopulations, across Ontario.

